# Risk of substance use disorders in the adult children of parents with severe alcohol use disorder: a nationwide cohort study

**DOI:** 10.1186/s12889-025-24900-9

**Published:** 2025-12-23

**Authors:** Kimberly Kane, Jeanette Westman, Johan Franck, Mika Gissler

**Affiliations:** 1 Department of Neurobiology, Care Sciences and Society, Karolinska Institutet, Stockholm, Sweden; 2 Region Stockholm, Academic Primary Health Care Centre, Box 45436, Stockholm, 104 31 Sweden; 3 Department of Clinical Neuroscience, Karolinska Institutet, Stockholm, Sweden; 4 Department of Molecular Medicine and Surgery, Karolinska Institutet, Stockholm, Sweden; 5 Department of Data and Analytics, Finnish Institute for Health and Welfare, Helsinki, Finland

**Keywords:** Adult children, Alcoholism, Epidemiology, Parents, Risk factors, Substance-Related disorders

## Abstract

**Background:**

Offspring of parents with alcohol use disorder (AUD) have elevated risk of substance use. However, few studies have comprehensively assessed risks associated with different substances. This study investigated the risk of substance use disorders (SUDs) in adult children with severe parental AUD over four decades, contributing information on the risk of each disorder, the roles of important risk factors, and the general versus substance-specific nature of SUD risk.

**Methods:**

Swedish national register data were used to follow children with and without ≥ 1 parent with an inpatient diagnosis of AUD from 1973 to 2018 to investigate risk of alcohol, opioid, cannabinoid, sedative/hypnotic, cocaine, other stimulant, hallucinogen, volatile solvent, and multiple drug use disorder. The composite outcomes any SUD, 1 SUD, and ≥ 2 SUDs including and excluding AUD were also investigated. Severe parental AUD and outcomes were defined with hospital inpatient diagnoses (ICD codes). Hazard ratios (HRs) were calculated with Cox regression. Model 1: unadjusted. Model 2: adjusted for child’s sex, parental education, and parental mortality. Model 3: Model 2 plus parental SUD. Model 4: Model 3 plus parental psychiatric disorder.

**Results:**

Risks of all outcomes were higher in those with (*n* = 99,723) than without (*n* = 2,321,756) severe parental AUD. For SUD diagnoses, the highest unadjusted risks were for other stimulant (HR 5.33, 95% CI 5.03–5.64), volatile solvent (HR 4.95, 95% CI 3.98–6.15), and opioid (HR 4.62, 95% CI 4.37–4.87) use disorders. After full adjustment, risks declined, and HRs of the different diagnoses converged to approximately twice as high in the adult children of parents with AUD. Risks of any SUD and of ≥ 2 SUDs were more elevated (95% CIs did not overlap) when AUD was included than when AUD was excluded. Risk of ≥ 2 SUDs was higher than risk of 1 SUD, but only when AUD was included.

**Conclusions:**

Severe parental AUD was associated with elevated risk for all SUDs. After full adjustment, SUD risks declined and converged but remained doubled. Sociodemographic factors, parental SUD, and parental psychiatric disorder explained much of the excess risk. Drug combinations that included alcohol elevated the risk of ≥ 2 SUDs and any SUD.

**Supplementary Information:**

The online version contains supplementary material available at 10.1186/s12889-025-24900-9.

## Introduction

Alcohol use disorder (AUD) is an important public health problem that impacts the person with the disorder and those around them, including their children [1]. The number of children who have a parent with AUD varies by place and time from approximately 2% to 23% [2, 3]. A substantial body of literature finds that the offspring of parents with AUD have an elevated risk for AUD [4] and for composite substance use disorder (SUD) outcomes [4–7]. Those findings are consistent with growing evidence that a common vulnerability underlies problematic use of a variety of substances, including but not limited to alcohol [8, 9]. Moreover, family [10] and family genetic [9] research suggests that AUD in families is associated with risk of other SUDs in a general rather than a substance-specific way. Few studies, however, have examined the risk of a broad range of specific substances other than alcohol in the offspring of parents with AUD. A study of the risk of specific SUDs in a national cohort of individuals with and without parental AUD can contribute information on the risk of each SUD, the roles of important factors in the risk of each disorder, and the general versus substance-specific nature of SUD risk.

Sweden’s national registers provide the opportunity to investigate risks of medically diagnosed SUDs in the adult children of parents with AUD. This is because children born in Sweden can be linked with their parents and with hospital diagnoses in both generations over a period of more than four decades. The comprehensive data in the registers also makes it possible to control for important factors such as sociodemographic characteristics and parental psychiatric disorder.

## Methods

### Aim

This national cohort study aimed to investigate the risk of substance use disorders in adult children of parents with severe alcohol use disorder.

### Study design, population, setting, and data sources

Data for this study came from Swedish national registers (Table 1). The Recording of Studies Conducted Using Observational Routinely Collected Health Data (RECORD) extension of the Strengthening the Reporting of Observational Studies in Epidemiology (STROBE) guidelines were used in preparing this manuscript [11].

**Table 1:** Sources and years of national register data used in the study

Register	Source	Data	Years
Medical Birth Register^1^	National Board of Health and Welfare	Maternity, child’s sex	1973–1995
Population Register	Statistics Sweden	Paternity	1973–1995
		Emigration	1973–2018
		Mortality	1973–2018
National Patient Register^2^	National Board of Health and Welfare	Disorders	1973–2018
Census	Statistics Sweden	Parental education	1970, 1975, 1980, 1985, 1990

^1^Live births

^2^Hospital inpatient diagnoses

The study cohort comprised all individuals recorded in the national Medical Birth Register as born in Sweden between January 1, 1973, and December 31, 1995. The Medical Birth Register was used to link 100% of the adult children to their mothers, and the Population Register to link 99% to their fathers. National Patient Register data were used to identify disorders in parents and to follow up diagnoses in the children through 2018. Because the children’s diagnoses were followed up into adulthood, the children are hereafter referred to as “adult children.”

Statistics Sweden, the Swedish national statistics agency, combined the data they hold (Population Register, Census) with data from the National Board of Health and Welfare (Medical Birth Register, National Patient Register) and replaced the personal identification number issued to each resident of Sweden with a code. Statistics Sweden delivered the combined data to M.G., who used the code to link the data in the different registers at the individual level. Three months after data delivery, Statistics Sweden destroyed the code key, rendering the data anonymous.

### Variables

The exposure variable in the study, severe parental AUD from nine months prior to the child’s birth until the child turned 18 years (yes/no), was defined using National Patient Register inpatient diagnoses in the form of codes from the World Health Organization’s International Statistical Classification of Diseases and Related Health Problems (ICD) (Additional file 1). Parents were classified as having severe AUD if they had a primary or secondary AUD-related hospital inpatient diagnosis from the 10th revision (ICD-10) or its historical Swedish ICD-8 and ICD-9 equivalents. ICD-10 codes used to indicate AUD were E24.4, F10, G31.2, G62.1, G72.1, I42.6, K29.2, K70, K85.2, K86.0, and O35.4. Two AUD-related codes found only in the Swedish version of the ICD-8, 261 and 262, were also included. Adult children were divided into those with severe parental AUD, defined as having at least one parent who received an AUD-related hospital inpatient diagnosis, and those without severe parental AUD (the reference group).

The outcome variables, SUDs in adult children (yes/no), were proxy measures defined using hospital inpatient diagnoses in the form of ICD-10 codes and their historical ICD-8 and ICD-9 equivalents from the National Patient Register (Additional files 1–10). Outcomes, listed by ICD-10 codes, included alcohol (F10; narrow definition), alcohol (as previously defined for parents, broad definition), opioid (F11), cannabinoid (F12), sedative or hypnotic (F13), cocaine (F14), other stimulant (F15), hallucinogen (F16), volatile solvent (F18), and multiple drug (F19) use disorders.

Several composite outcomes consisting of ICD-10 codes and their ICD-8 and ICD-9 equivalents were included in the study to examine the risks of any SUD and of one vs. multiple SUDs including and excluding AUD. These were “any SUD,” “any SUD excluding AUD,” “one SUD,” “one SUD excluding AUD,” “two or more SUDs,” and “two or more SUDs excluding AUD.” ICD codes used to define the outcomes are provided in the results table that shows the number and proportion diagnosed with each outcome. The multiple SUD outcomes were added because it is unclear how physicians in Sweden interpreted and used ICD-10 code F19 (multiple drug use disorder) and its historical equivalents during the study period. The Swedish ICD-10 clarifies that the category should be employed when a person used two or more substances but the main substance was unknown or the substances used could not be clearly determined [12]. The definition does not explicitly include or exclude alcohol.

Data on SUDs in adult children were collected from birth to emigration, death, or the end of the study period, whichever came first.

The highest level of parental education, a proxy for socioeconomic status, was divided into basic (≤ 9 years), secondary (10 − 12 years), tertiary (≥ 13 years), and missing. The variable measured the highest level of education achieved by the mother or father, whichever was higher, and came from the 1970, 1975, 1980, 1985, and 1990 Swedish census.

Information on the death of at least one parent before the child turned 18 years (yes/no), came from the Population Register for 1973 through 2018. Parental mortality is associated with negative outcomes in children, such as lower levels of education and employment, poor mental health, and SUDs [13].

Data on parental SUD and parental psychiatric disorder (yes/no) came from the National Patient Register for 1973 through 2018. Parental SUD was defined as at least one diagnosis of any SUD other than AUD in at least one parent. Parental psychiatric disorder was defined as at least one diagnosis from Chapter V of the ICD-8, ICD-9, or ICD-10, in at least one parent, excluding AUD and other SUDs. The excluded diagnoses were ICD-10 codes F10–F19; ICD-9 codes 291–292 and 303–305; and ICD-8 codes 291 and 303–304.

Information on emigration and mortality came from the Population Register for the years 1973 through 2018. Data on the sex of the adult children (female/male) came from the Medical Birth Register for the years 1973 through 1995.

### Statistical analyses

Chi-square tests were used to investigate differences between categorical background variables. Cox regression models were used to estimate hazard ratios (HRs) and 95% confidence intervals (CIs) of SUDs in adult children with and without severe parental AUD. Cox regression was chosen for its ability to account for the actual time individuals were at risk during the study period and because it allows the hazard of the outcome to vary flexibly over time. Calendar time was used as the time scale in the Cox model. Trends for exposed and unexposed individuals over time were examined visually. They were similar and thus consistent with the proportional hazards assumption. The crude model (Model 1) included only severe parental AUD (yes/no). Model 2 was adjusted for the adult child’s sex, parental education, and death of at least one parent before the child turned 18 years. Model 3 was adjusted for the same factors as Model 2 plus parental SUD. Model 4 was adjusted for the same factors as Model 3 plus parental psychiatric disorder. *P* values < 0.05 were considered statistically significant.

SAS/STAT software, Version 9.4 of the SAS System for Windows 10 (SAS Institute Inc., Cary, NC, USA), and Stata statistical software, Version 17.0 (StataCorp LLC, College Station, TX, USA), were used in the analyses.

## Results

### Characteristics of the study population

A total of 4.1% (*n* = 99,723) of the cohort had at least one parent with severe AUD and 95.9% (*n* = 2,321,756) did not (Table 2). Significantly more parents with than without severe AUD had a basic (31.8% versus 21.0%) or secondary education (34.3% versus 33.2%) or were missing data on their education (16.7% versus 8.0%) (*P* < 0.001). Those with severe parental AUD were more likely to have lost a parent before turning 18 than those without severe parental AUD (14.5% versus 2.9%, *P* < 0.001).

**Table 2:** Characteristics of the study population^1,2^

		Total study population	Without severe parental alcohol use disorder^3^	With severe parental alcohol use disorder^3^	*P* value^4^
Number (% of total study population)		2,421,479 (100)	2,321,756 (95.9)	99,723 (4.1)	
Sex, n (%)					0.64
Male		1,244,135 (51.4)	1,192,970 (51.4)	51,165 (51.3)	
Female		1,177,344 (48.6)	1,128,786 (48.6)	48,558 (48.7)	
Highest level of parental education, n (%)					
Basic (≤ 9 years)		520,057 (21.5)	488,309 (21.0)	31,748 (31.8)	*<* 0.001
Secondary (10 − 12 years)		804,441 (33.2)	770,269 (33.2)	34,172 (34.3)	*<* 0.001
Tertiary (≥ 13 years)		893,791 (36.9)	876,651 (37.8)	17,140 (17.2)	*<* 0.001
Missing		203,190 (8.4)	186,527 (8.0)	16,663 (16.7)	*<* 0.001
One or both parents died before the child turned 18 years, n (%)		82,122 (3.4)	67,638 (2.9)	14,484 (14.5)	*<* 0.001

^1^The study population is the same as the study population in a previous study from the research group. This table is adapted from Table 1 in that article, Kane K, Westman J, Franck J, Gissler M. Risk of severe mood and anxiety disorders in the adult children of parents with alcohol use disorder: a nationwide cohort study. J Epidemiol Community Health. 2024 Jun 10;78(7):444–450. doi: 10.1136/jech-2023-221720. PMID: 38,688,702; PMCID: PMC11187371 under the terms of the CC BY-NC 4.0 license: https://creativecommons.org/licenses/by/4.0/ [14]

^2^The 2,421,479 children born in Sweden (live births) between 1 January 1973 and 31 December 1995

^3^See Additional file 1 for the International Statistical Classification of Diseases and Related Health Problems (ICD) codes used to define severe parental alcohol use disorder in the study. The study included parental inpatient hospital diagnoses of alcohol-related disorders received from nine months before the child was born and until the child turned 18

^4^For the difference between those with and without severe parental AUD. Severe parental AUD was defined as having at least one parent with a hospital inpatient diagnosis of an AUD-related disorder

### Proportions diagnosed

A higher proportion of adult children with than without severe parental AUD had every outcome in the study (Table 3). This was true for specific SUD diagnoses (e.g., opioid use disorder: with severe parental AUD, *n* = 1559, 1.6%; without severe parental AUD, *n* = 7928, 0.3%; *P* < 0.001) and composite outcomes (e.g., any SUD: with severe parental AUD, *n* = 11,130, 11.2%; without severe parental AUD, *n* = 80,970, 3.5%; *P* < 0.001).

**Table 3:** Number and proportion diagnosed with substance use disorders^1,2^ in the study population^3^

	All	Without severe parental AUD	With severe parental AUD	*P* value^4^
Number (% of total study population)	2,421,479 (100)	2,321,756 (95.9)	99,723 (4.1)	< 0.001
Number with the composite outcome (%)				
Any substance use disorder				
One or more ICD-10 codes F10-F16, F18-F19, as well as the ICD-8/ICD-9 equivalents	92,100 (3.8)	80,970 (3.5)	11,130 (11.2)	< 0.001
Any substance use disorder excluding AUD				
One or more ICD-10 codes F11-F16, F18, as well as the ICD-8/ICD-9 equivalents	10,921 (0.5)	9724 (0.4)	1197 (1.2)	< 0.001
One substance use disorder				
One ICD-10 code F10-F16 or F18 as well as the ICD-8/ICD-9 equivalents	58,138 (2.4)	52,371 (2.3)	5767 (5.8)	< 0.001
One substance use disorder excluding AUD				
One ICD-10 code F11-F16, F18 as well as the ICD-8/ICD-9 equivalents	9453 (0.4)	8442 (0.4)	1011 (1.0)	< 0.001
Two or more substance use disorders				
F19 or two or more of ICD-10 codes F10-F16, F18 as well as the ICD-8/ICD-9 equivalents	33,962 (1.4)	28,599 (1.2)	5363 (5.4)	< 0.001
Two or more substance use disorders excluding AUD				
Two or more of ICD-10 codes F11-F16, F18, as well as the ICD-8/ICD-9 equivalents	1468 (0.1)	1282 (0.1)	186 (0.2)	< 0.001
Number with the inpatient hospital diagnosis (%)				
Alcohol use disorder (including alcohol-related diagnoses)				
ICD-10: F10 or other alcohol-related diagnoses and the ICD-8/ICD-9 equivalents^5^	63,108 (2.6)	55,769 (2.4)	7339 (7.4)	< 0.001
Alcohol use disorder				
ICD-10: F10 and the ICD-8/ICD-9 equivalents^6^	62,441 (2.6)	55,181 (2.4)	7260 (7.3)	< 0.001
Multiple drug use disorder				
ICD-10: F19 and the ICD-8/ICD-9 equivalents^2^	28,467 (1.2)	23,857 (1.0)	4610 (4.6)	< 0.001
Sedative or hypnotic use disorder				
ICD-10: F13 and ICD-8/ICD-9 equivalents^2^	11,749 (0.5)	9989 (0.4)	1760 (1.8)	< 0.001
Opioid use disorder				
ICD-10: F11 and ICD-8/ICD-9 equivalents^2^	9487 (0.4)	7928 (0.3)	1559 (1.6)	< 0.001
Cannabinoid use disorder				
ICD-10: F12 and ICD-8/ICD-9 equivalents^2^	9049 (0.4)	7750 (0.3)	1299 (1.3)	< 0.001
Other stimulant use disorder				
ICD-10: F15 and ICD-8/ICD-9 equivalents^2^	7730 (0.3)	6301 (0.3)	1429 (1.4)	< 0.001
Hallucinogen use disorder				
ICD-10: F16 and ICD-8/ICD-9 equivalents^2^	1553 (0.1)	1313 (0.1)	240 (0.2)	< 0.001
Cocaine use disorder				
ICD-10: F14 and ICD-8/ICD-9 equivalents^2^	1278 (0.1)	1095 (0.0)	183 (0.2)	< 0.001
Volatile solvent use disorder				
ICD-10: F18 and ICD-8/ICD-9 equivalents^2^	568 (0.0)	469 (0.0)	99 (0.1)	< 0.001

*AUD* Alcohol use disorder, *ICD* International Statistical Classification of Diseases and Related Health Problems, *ICD*-*10* 10th revision of the ICD, *ICD*-*9* 9th revision of the ICD, *ICD*-*8* 8th revision of the ICD

^1^Substance use disorders listed by highest to lowest frequency in the adult children without severe parental AUD

^2^See Additional files 1–10 for list of ICD codes used to define the variables in the study. The study included parental inpatient hospital diagnoses of alcohol-related disorders received from 9 months before the child was born and until the child turned 18

^3^The 2,421,479 children born in Sweden (live births) between 1 January 1973 and 31 December 1995

^4^For the difference between those with and without severe parental AUD. Severe parental AUD was defined as having at least one parent with a hospital inpatient diagnosis of an AUD-related disorder

^5^This row presents results for severe AUD in adult children defined using the broader list of AUD-related ICD codes that were used to define severe parental AUD (see Additional file 1)

^6^This row presents the results for severe AUD in adult children defined as ICD-10 code F10 and its historical ICD-8 and ICD-9 equivalents (see Additional file 2)

AUD was the most common SUD diagnosis regardless of whether severe parental AUD was present. For example, 7260 (7.3%) of those with severe parental AUD had a hospital diagnosis of AUD, whereas 1760 (1.8%) had a hospital diagnosis of sedative or hypnotic use disorder. Regardless of parental AUD, the majority of individuals with any SUD had AUD either alone or in combination with other disorders (e.g., *n* = 81,079 or 88.1% of those in the total study population with any SUD). Similarly, regardless of parental AUD, more than 80% of those with one SUD had AUD (e.g., *n* = 48,685 or 83.7% of individuals with one SUD in the total study population). Alcohol was a part of almost all combinations of two or more SUDs, again regardless of parental AUD (e.g., *n* = 32,494 or 95.7% of individuals with two or more SUDs in the total study population).

The number of adult children with severe AUD increased by 667 when AUD in adult children was defined with AUD-related ICD codes (broader definition) rather than with ICD-10 code F10 and its historical ICD-8 and ICD-9 equivalents (narrower definition) (severe parental AUD, *n* = 7339, 7.4%; no severe parental AUD *n* = 55,769, 2.4%) (*P* < 0.001) (Table 3). In adult children with and without severe parental AUD, the most common individual SUD diagnoses other than AUD were multiple drug, sedative or hypnotic, and opioid use disorders. The least common was volatile solvent use disorder.

### Risks (hazard ratios) of any SUD and individual SUDs

**Fig. 1:**
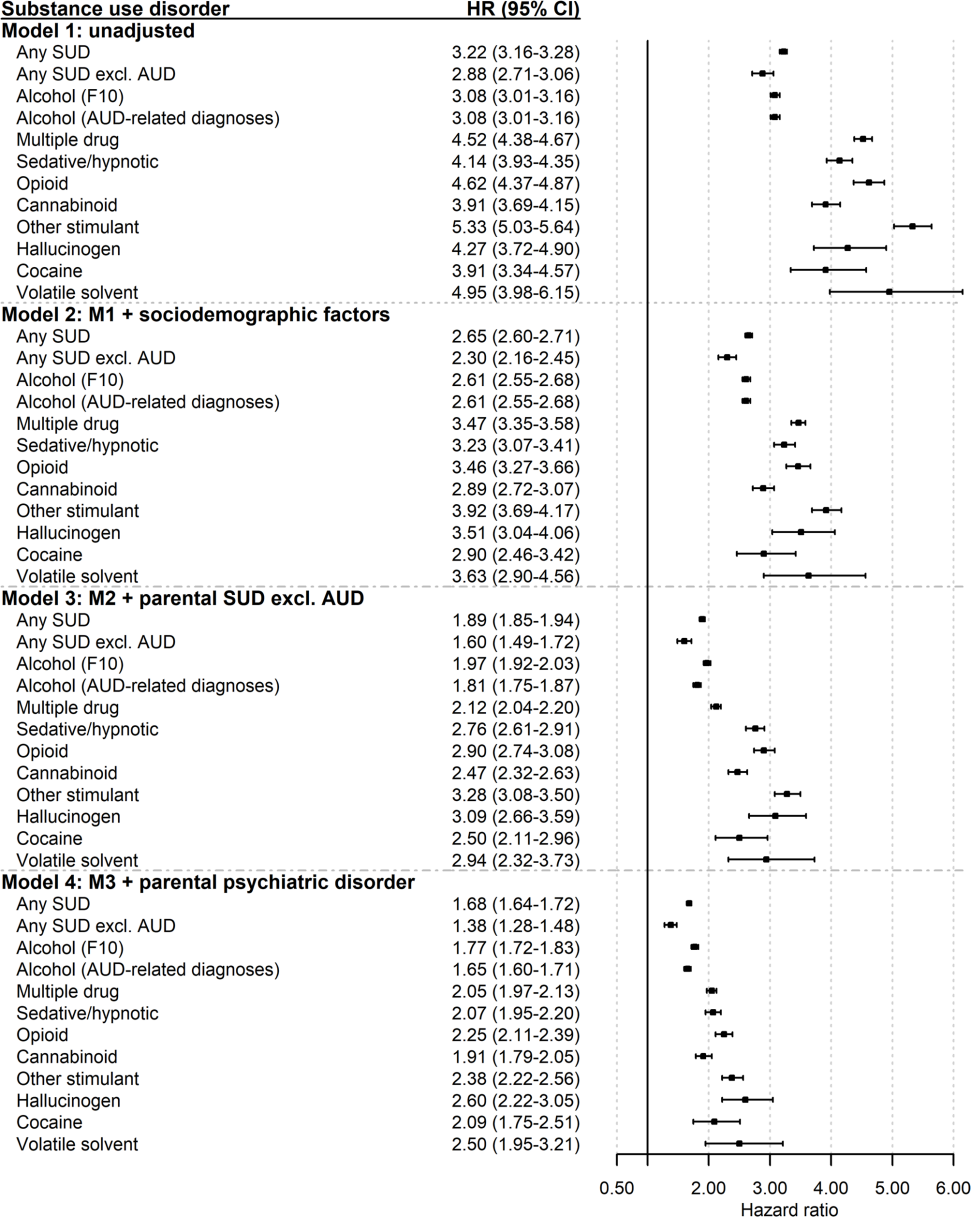
Crude and adjusted hazard ratios and 95% confidence intervals of any *SUD* and specific *SUD *diagnoses in adult children with at least one parent with severe *AUD* in Sweden between 1973 and 2018 (*n* = 99,723). The reference group was adult children of parents without severe *AUD *(*n* = 2,321,756). The crude model (Model 1) was unadjusted. Model 2 was adjusted for child’s sex, parents’ highest level of education, and death of a parent before the child turned 18 years. Model 3 was adjusted for the same variables as Model 2 plus parental substance use disorder. Model 4 was adjusted for the same variables as Model 3 plus parental psychiatric disorder. *Abbreviations*: *AUD* Alcohol use disorder, *AUD*-related diagnoses, severe *AUD *in adult children defined using a broader list of *AUD*-related *ICD *codes (see Additional file 1), *CI* Confidence interval, *excl*., excluding, F10, severe *AUD *in adult children defined using a narrower list of ICD codes (see Additional file 2), *HR* Hazard ratio, *ICD* International Statistical Classification of Diseases and Related Health Problems, *M* Model, *SUD* Substance use disorder

In all models, risks of each SUD diagnosis and of any SUD were elevated in the adult children with severe parental AUD (Fig. 1). Risks of any SUD were statistically meaningfully higher (95% CIs did not overlap) when AUD was included in the variable than when it was not (Additional file 11).

In Model 1, the crude (unadjusted) model, severe parental AUD was associated with an approximately 3-fold risk of AUD (HR 3.08, 95% CI 3.01–3.16) and of any SUD regardless of whether AUD was included in the composite variable (any SUD: HR 3.22, 95% CI 3.16–3.28; any SUD excluding AUD: HR 2.88, 95% CI 2.71–3.06) (Fig. 1). Adult children with severe parental AUD had 4 to 5 times higher risk of other specific SUDs. Risks were most elevated for other stimulant, volatile solvent, opioid, and multiple drug use disorders (e.g., multiple drug: HR 4.52, 95% CI 4.38–4.67).

After adjustment for sociodemographic factors in Model 2, the elevation in risks of all outcomes declined. The risk of any SUD and of AUD declined to approximately 2.5 times that of adult children without parental AUD (any SUD: HR 2.65, 95% CI 2.60–2.71; AUD: HR 2.61, 95% CI 2.55–2.68). When AUD diagnoses were excluded from “any SUD,” adjustment for sociodemographic factors resulted in a risk just over 2 times that of adult children without severe parental AUD (HR 2.30, 95% CI 2.16–2.45). The risk of other SUDs declined to approximately 3 to 4 times higher (e.g., multiple drug: HR 3.47, 95% CI 3.35–3.58). Particularly large declines were observed for other stimulant and volatile solvent use disorders.

Following additional adjustment for parental SUD in Model 3, excess risk of most outcomes declined to between approximately 2 to 3.5 times higher in adult children with than without severe parental AUD (Fig. 1). A particularly large decline was observed for multiple drug use disorder (HR 2.12, 95% CI 2.04–2.20). For AUD and for any SUD, excess risk declined to slightly less than 2 times higher in the adult children with severe parental AUD (e.g., any SUD: HR 1.89, 95% CI 1.85–1.94). The reduction in excess AUD risk was greater in the additional analysis that used the broader definition of adult children’s AUD than in the analysis that used the narrower definition.

After full adjustment for sociodemographic factors, parental SUD, and parental psychiatric disorder in Model 4, risks of any SUD and the specific SUD diagnoses again declined. Most remained approximately 2 to 2.5 times higher in adult children with severe parental AUD. Notable exceptions were any SUD and AUD, for which risk was elevated but was less than 2 times higher in adult children with severe parental AUD (e.g., any SUD: HR 1.68, 95% CI 1.64–1.72). A particularly small decline was observed for multiple drug use disorder, from HR 2.12 (95% CI 2.04–2.20) to HR 2.05 (95% CI 1.97–2.13). The highest excess risks were for hallucinogen, volatile solvent, other stimulant, and opioid use disorders.

### Risks (hazard ratios) of one vs. two or more SUDs

**Fig. 2:**
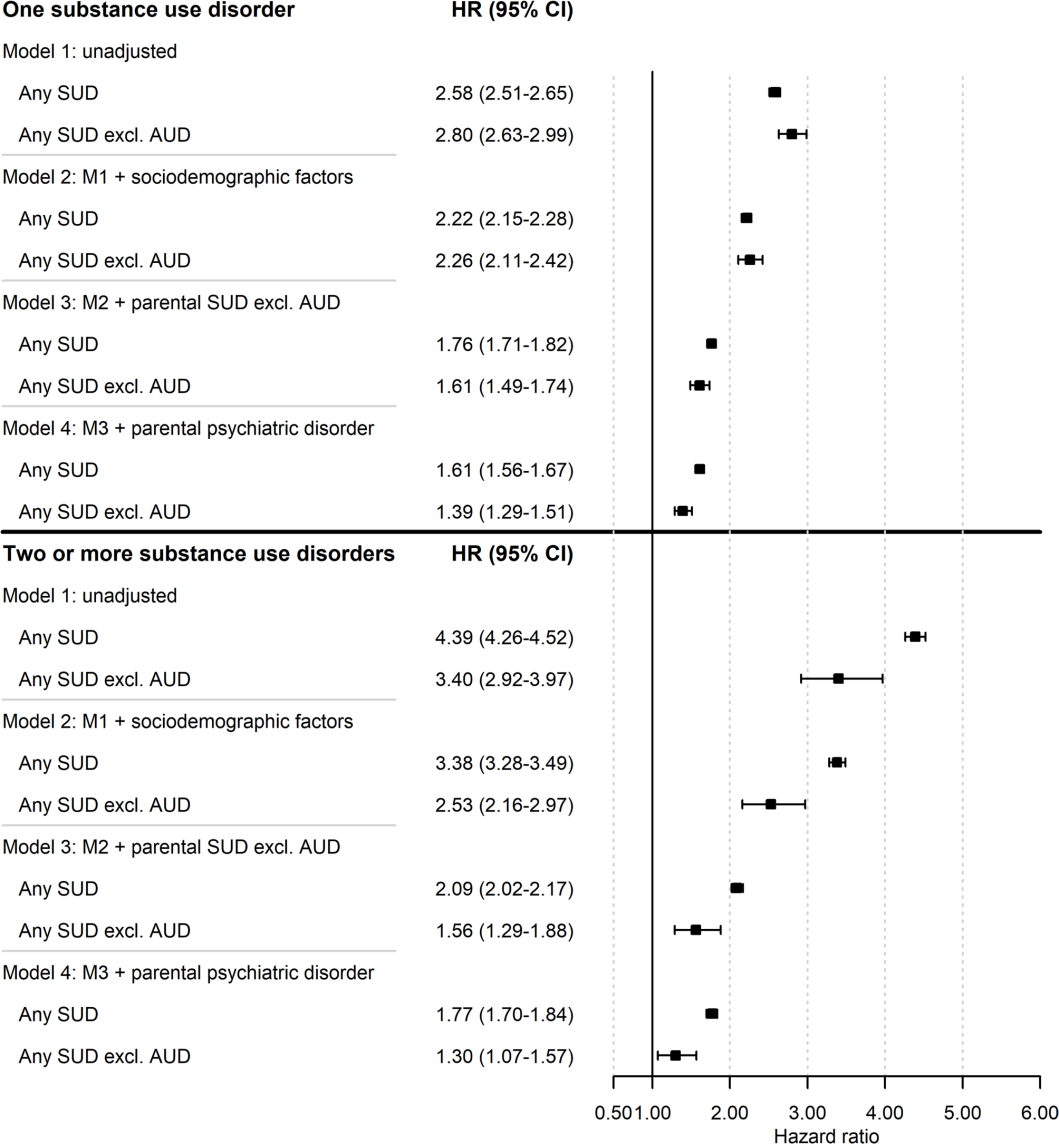
Crude and adjusted hazard ratios and 95% confidence intervals of one SUD and two or more SUDs, including and excluding AUD in adult children with at least one parent with severe AUD in Sweden between 1973 and 2018 (*n* = 99,723). The reference group was adult children of parents without severe AUD (*n* = 2,321,756). The crude model (Model 1) was unadjusted. Model 2 was adjusted for child’s sex, parents’ highest level of education, and death of a parent before the child turned 18 years. Model 3 was adjusted for the same variables as Model 2 plus parental substance use disorder. Model 4 was adjusted for the same variables as Model 3 plus parental psychiatric disorder. *Abbreviations*:* AUD* Alcohol use disorder, *AUD*-related diagnoses, severe AUD in adult children defined using a broader list of AUD-related ICD codes (see Additional file 1), *CI* Confidence interval, *excl*. Excluding, F10, severe AUD in adult children defined using a narrower list of ICD codes (see Additional file 2), *HR* Hazard ratio, *ICD* International Statistical Classification of Diseases and Related Health Problems, *M* Model, *SUD* Substance use disorder

The risk of one SUD and of two or more SUDs was elevated in the adult children with severe parental AUD in all models (Fig. 2). Risks declined after each sequential adjustment for potential explanatory variables. For those with two or more SUDs, the most substantial decline was observed after the additional adjustment for parental SUD other than AUD (Additional file 11). In most instances, the declines between models were statistically meaningful (95% CIs did not overlap between models). However, for adult children with two or more SUDs excluding AUD, the only sequentially included variable that was clearly statistically meaningful was parental SUD other than AUD.

The inclusion of AUD made a difference in the risk of one SUD and of two or more SUDs in the adult children with severe parental AUD but was more important to two or more SUDs (Additional file 11). Like the risk of any SUD, the risk of two or more SUDs was statistically meaningfully higher in all models when AUD was included than when it was excluded (e.g., Model 1: including AUD, HR 4.39, 95% CI 4.26–4.52 vs. excluding AUD, HR 3.40, 95% CI 2.92–3.97). The same was not true of the risk of one SUD, where the inclusion of AUD made a statistically meaningful difference only in the final model.

The excess risk of two or more SUDs was higher than the excess risk of one SUD (Additional file 12). However, the difference was statistically meaningful only when AUD was included in the outcome. For example, in the unadjusted model that included AUD, the HR for two or more SUDs was 4.39 (95% CI 4.26–4.52) and for 1 SUD, 2.58 (95% CI 2.51–2.65). When AUD was excluded, the HR for two or more SUDs was 3.40 (95% CI 2.92–3.97) and for one SUD, 2.80 (95% CI 2.63–2.99).

## Discussion

This study on the risks of SUDs in adult children with severe parental AUD found that the risks of specific SUDs (e.g., opioid use disorder, cocaine use disorder) and of composite SUD outcomes (any, one, or two or more SUDs) were elevated in adult children with severe parental AUD. The highest excess risks of specific SUDs were for other stimulant, volatile solvent, opioid, and multiple drug use disorders. After adjusting for sociodemographic factors, parental SUD, and parental psychiatric disorder in the final model, risks of specific SUDs declined, and HRs of the diagnoses converged to approximately twice as high in the adult children of parents with than without severe AUD. This convergence suggests that excess risk of SUDs is similar across different SUD diagnoses. Factors that stood out as particularly influential in specific outcomes included sociodemographic factors in the increased risk of stimulant use disorder, as well as parental SUDs other than AUD in the increased risk of multiple SUDs. The findings on composite SUD outcomes showed that drug combinations that included alcohol played a noteworthy role in the excess risk of multiple SUDs and, by extension, any SUD. It is worth highlighting that despite their elevated risk, the great majority of adult children with severe parental AUD remained free of diagnoses of even the most common SUDs, including AUD.

With regard to the current study’s findings on incidence, in keeping with prior research [15], AUD was the most common SUD diagnosis in the study population. Multiple drug use disorder (ICD-10 code F19) was also common in the cohort, as was the composite variable two or more SUDs. The frequency of multiple SUDs in the study population is consistent with findings that multiple substance use is common in people treated for SUDs [16, 17].

Turning to the findings on hazard ratios, the elevated risk of SUDs observed in the adult children with severe parental AUD was in line with the results of previous research [4–7], and the finding that risks of specific SUDs converged in the final model was consistent with the hypothesis that in families with AUD, the risk for non-alcohol SUDs is general rather than substance specific. Most prior research on this topic comes from studies that examined family risk factors and genetics. For example, one study of 803 individuals and their families found that while family AUD predicted AUD in individuals, it was also a consistent and non-specific predictor of other SUDs [10]. A slightly different result was observed in a family genetic study from Sweden [9]. That study found minor specific genetic risk for certain SUDs, but for the most part, SUDs, including AUD, were associated with the risk of other SUDs in a non-specific way. The hypothesis that SUD risk has an important general component is also supported by genome-wide association studies that have observed evidence of a general addiction risk factor for alcohol, tobacco, cannabis, and opioids [18, 19] possibly related to dopamine regulation [18]. Researchers have suggested that a general genetic liability may express itself as use of specific substances based on the availability and acceptability of those substances [20], and the findings of the current study are consistent with that hypothesis.

Other stimulant use disorder was the disorder for which risk was most elevated in those whose parents had severe AUD. Amphetamines and methamphetamines have been used in Sweden since the late 1930s, and use reached a peak in the 1950s [21]. The drugs remained common into the 2000s, more so than in many other European countries [22]. The present study results are consistent with those of a 2009 investigation of Swedish Prison and Probation Service clients who were primary users of amphetamines, heroin, and cocaine [22]. That study found that amphetamines were the most common of the three and that parental alcohol problems were more characteristic of the amphetamine users. In the current study, adjustment for sociodemographic factors had an important impact on the risk of other stimulant use disorder, which is consistent with the history of amphetamine use in Sweden and with research from the United States that found that illicit stimulant use is associated with low socioeconomic status [23].

Another robust finding was the elevated risk of opioid use disorder in the adult children with severe parental AUD. This result is broadly consistent with those in the limited literature on the connection between parental AUD and risk of opioid use disorder in the next generation. For example, a study of 87 treatment-resistant opioid users found that parental use of alcohol was associated with childhood maltreatment, which in turn was associated with starting to inject opioids at a younger age [24]. Furthermore, an analysis of U.S. national survey data connected parental alcohol use with adolescent children’s nonmedical use of prescription opioids, an association that disappeared after adjustment for potential confounders [25]. Finally, another study based on U.S. national survey data found that a history of parental alcoholism significantly increased the odds of prescription drug misuse and disorder in a subpopulation of people who self-identified as Latino [26]. The prescription drug outcome was broad and covered opioids, sedatives, and tranquilizers.

The elevated risk of multiple SUDs, observed for all proxy measures used in the study, was consistent with the increased risk of SUDs in general in people with parental AUD [4–7]. Few studies have examined parental AUD and risk of multiple SUDs in adult children. However, latent-class analysis of data from a U.S. national survey found that parental drinking increased the risk of being in the class with the highest probability of polysubstance use [27].

A result that stood out was the substantial decline in excess risk of all measures of multiple SUDs after adjustment for parental SUD in addition to AUD. Declines were similar regardless of whether the multiple SUD outcome included AUD. The finding indicates that a parent’s dependence on alcohol and at least one other substance is a warning sign that their children are vulnerable to SUDs. The relatively small decline after additional adjustment for parental psychiatric disorder may be explained by high psychiatric comorbidity in people with AUD and other SUDs [1].

The risk of multiple SUDs was meaningfully higher than that of single SUDs only when AUD was included in the outcomes. Thus, alcohol was important in substance combinations in those with severe parental AUD. The finding also suggests that combinations of alcohol and other substances played an important role in the raised risk of two or more SUDs, and by extension, in the overarching category of any SUD. These results are consistent with existing evidence that the offspring of parents with AUD have an elevated risk of AUD [4], and that individuals with one SUD have an increased risk of developing additional SUDs [28].

Cannabis is the most-used illicit drug in Sweden [29] and worldwide [30]. Numerous studies have found that parental alcohol use is associated with cannabis use-related outcomes in the next generation [31–33]. Fewer studies have investigated cannabis-related disorders as outcomes, but researchers who followed nearly 2500 children born in the early 1980s in Brisbane, Australia, for over 20 years found that those whose mothers drank >1 glass of alcohol a day had higher odds of cannabis use disorder than those whose mothers drank ≤ 1 glass a day [34]. Additionally, a cross-sectional study of more than 3000 college students in northeastern France observed a connection between parental alcohol dependence and young adults’ cannabis dependence [35]. However, in an interview study of 719 young adults and their parents in the U.S. state of Oregon, no association was seen between parental AUD and cannabis use disorder [36].

There are few prior studies on the risk of sedative or hypnotic use disorder in the adult children of parents with AUD, so the information provided here is novel. Consistent with the current findings of elevated risk in the adult children with severe parental AUD, a U.S. national survey study found that a history of parental alcoholism significantly increased the odds of prescription drug misuse and prescription drug use disorder, a composite measure that covered sedatives, tranquilizers, and opioids [26].

The risk of cocaine use disorder was elevated in the adult children with severe parental AUD but was not among the most elevated risks in the study. Because patterns of cocaine use differ in Sweden and elsewhere, caution is needed in generalizing the findings to other contexts. Historically, cocaine use has been less common in Sweden than in the United States and elsewhere in Europe, and cocaine has primarily been a club drug used by people under the age of 40 [37].

There are few specific studies on the association between parental AUD and cocaine use disorder in the next generation. In line with the current findings, however, a 1990s study of more than 400 male adolescents incarcerated in a large U.S. city suggested a relationship between parental alcohol problems and cocaine use disorder in the participants [38].

This cohort study, like others, provides information about risk, but causality cannot be inferred. The strengths and limitations common to national register studies are also found in the current study. Individual-level, linkable information was available on the national population, including health outcomes and sociodemographic factors. Additionally, the accuracy of the diagnoses in the National Patient Register is generally high [39, 40]. Inpatient care coverage is nearly complete, although data are missing for a few of Sweden’s counties for specific years in the mid-1980s [41].

As in other national register studies, however, the data were not collected specifically for the study, so some information of interest is missing. For example, although parental mortality data were available to the researchers, information on separation from parents for other reasons or data on the children’s living situations was not. A further limitation of the registers used in the study is that they do not include information from social services or self-reported data such as adverse childhood experiences or protective factors in the children’s lives. Additionally, data on children born outside Sweden were not available to the researchers.

Several limitations were specific to the use of inpatient data from the National Patient Register to define AUD and other psychiatric disorders. Most parental AUD identified in the register was probably on the severe end of the spectrum. This is the case even though both primary and secondary diagnoses of AUD-related disorders were used in the study, so AUD was not necessarily the main reason for every hospital visit that resulted in a diagnosis. Moreover, the researchers did not have access to data on psychiatric outpatient or primary care diagnoses, convictions, prescriptions, or participation in psychosocial treatment (e.g., 12-step programs). The study therefore underestimated the total number of people with parental AUD, and the results are less generalizable to and may overestimate SUD risk in the many people whose parents have mild or moderate AUD. Furthermore, certain characteristics affect the likelihood that a person with AUD will appear in different Swedish national registers, possibly because they affect behaviors such as care-seeking [42]. For this reason, women, younger people, and people with higher levels of education with AUD may have been underrepresented in the data. The results may be most relevant to places that have similar sociodemographic characteristics, patterns of substance use, and social and health care systems.

The study was exploratory, and the findings can be vulnerable to the multiple comparisons problem even though the data are on the whole population. Additionally, the findings estimate the average association between severe parental AUD and SUDs during the study period. Analyses of time trends over the study period were beyond the scope of this study.

## Conclusion

Adult children with severe parental AUD had about three times the risk of AUD and four to five times the risk of every other SUD than those without parental AUD, including opioid, cannabinoid, sedative or hypnotic, cocaine, other stimulant, hallucinogen, volatile solvent, and multiple drug use disorders. The composite outcomes of any, one, and two or more SUDs were also elevated in adult children with severe parental AUD. Risks of specific SUDs declined after adjustment for important risk factors, including the adult child’s sex, highest level of parental education, death of a parent, parental SUD, and parental psychiatric disorder. However, they remained approximately two times higher in adult children with than without severe parental AUD. The results thus show a similar pattern of elevated risk for specific SUDs in adult children with severe parental AUD. The findings highlight the importance of sociodemographic factors in the increased risk of stimulant use disorder and of parental SUDs in addition to AUD in the increased risk of multiple SUDs. Additionally, the results show that drug combinations that include alcohol elevate the excess risk of multiple SUDs and of any SUD.

## Supplementary Information

Supplementary Material 1.

## Data Availability

The data used in this study came from the Swedish National Board of Health and Welfare and Statistics Sweden. Because of data protection regulations, they are not publicly available. To apply for these or similar data, contact the National Board of Health and Welfare and Statistics Sweden.

## Abbreviations

AUD: Alcohol use disorder
CI: Confidence interval
HR: Hazard ratio
ICD: International Statistical Classification of Diseases and Related Health Problems
SUD: Substance use disorder
