# Supplementary material for: Risk of substance use disorders in the adult children of parents with severe alcohol use disorder: a nationwide cohort study
Source: BMC Public Health. 2025 Dec 23;25:4277. doi: 10.1186/s12889-025-24900-9 (PMC12723945; doi:10.1186/s12889-025-24900-9)
Supplement: Supplementary file 1 — Supplementary Material 1. [file 12889_2025_24900_MOESM1_ESM.docx]

# Risk of substance use disorders in the adult children of parents with severe alcohol use disorder: a nationwide cohort study

## Additional files

| **Additional file 1.** ICD codes used to define AUD-related disorders (the broader definition of AUD used in the study) | | | |
| --- | --- | --- | --- |
| **ICD-10** |  |  |  |
| E24.4 | Alcohol-induced pseudo-Cushing syndrome |  |  |
| F10 | Mental and behavioral disorders due to use of alcohol |  |  |
| G31.2 | Degeneration of nervous system due to alcohol |  |  |
| G62.1 | Alcoholic polyneuropathy |  |  |
| G72.1 | Alcoholic myopathy |  |  |
| I42.6 | Alcoholic cardiomyopathy |  |  |
| K29.2 | Alcoholic gastritis |  |  |
| K70 | Alcoholic liver disease |  |  |
| K85.2 | Alcohol-induced acute pancreatitis |  |  |
| K86.0 | Alcohol-induced chronic pancreatitis |  |  |
| O35.4 | Maternal care for (suspected) damage to fetus from alcohol |  |  |
| **ICD-9** |  |  |  |
| 291A | Delirium tremens | 291.0 | Delirium tremens |
| 291B | Korsakovs psykos, alkoholbetingad | 291.1 | Korsakov’s psychosis, alcoholic |
| 291C | Alkoholbetingad demens annan än 291A-B | 291.2 | Other alcoholic dementia |
| 291D | Alkoholhallucinos | 291.3 | Alcoholic hallucinosis |
| 291E | Patologiskt alkoholrus | 291.4 | Pathological drunkenness |
| 291F | Patologisk svartsjuka [alkohol relaterat] | 291.5 | Alcoholic jealousy (OR Pathological alcohol-related jealousy) |
| 291W | Specificerad alkoholpsykos annan än 291A-F | 291.8 | Other alcoholic psychosis |
| 291X | Alkoholpsykoser, ospecificerad | 291.9 | Alcoholic psychoses, unspecified |
| 305A | Alkoholmissbruk | 305.0 | Nondependent abuse of alcohol |
| 303X | Alkoholberoende | 303 | Alcohol dependence syndrome |
| 357F | Alkoholpolyneuropati | 357.5 | Alcoholic polyneuropathy |
| 425F | Alkoholkardiomyopati | 475.8 | Alcoholic cardiomyopathy |
| 535D | Gastrit orsakad av alkohol | 535.3 | Alcoholic gastritis |
| 571A | Alkoholfettlever | 571.0 | Alcoholic fatty liver |
| 571B | Akut alkoholhepatit | 571.1 | Acute alcoholic hepatitis |
| 571C | Levercirros orsakad av alcohol | 571.2 | Alcoholic cirrhosis of liver or Laennec's cirrhosis |
| 571D | Ospecificerad leverskada orsakad av alkohol | 571.3 | Alcoholic liver damage, unspecified |
| 977D | Förgiftning med medel vid alkoholavvänjning | 977.3 | Poisoning by other and unspecified drugs and medicaments, Alcohol deterrents |
| **ICD-8** |  |  |  |
| 261,00 | Beriberi alcoholic | 261 | Thiamine deficiency^1^ |
| 262,00 | Pellagra alcoholic | 262 | Niacin deficiency^1^ |
| 291,00 | Delirium tremens | 291.0 | Delirium tremens |
| 291,10 | Psychosis korsakow (alcoholica) | 291.1 | Korsakov’s psychosis (alcoholic) |
| 291,20 | Hallucinosis alcoholica alia | 291.2 | Other alcoholic hallucinosis |
| 291,30 | Paranoia alcoholica | 291.3 | Alcoholic paranoia |
| 291,98 | Psychosis alcoholica alia definita | 291.9 | Other and unspecified alcoholic psychoses^2^ |
| 291,99 | Psychosis alcoholica nud |  |  |
| 303,00 | Alcoholismus episodicus | 303.0 | Episodic excessive drinking |
| 303,10 | Alcoholismus habitualis | 303.1 | Habitual excessive drinking |
| 303,20 | Alcoholismus chronicus ("alcoholic addiction") (dipsomania) | 302.2 | Alcohol addiction |
| 303,98 | Alcoholismus alius definitus | 303.9 | Other and unspecified alcoholism^3^ |
| 303,99 | Alcoholismus nud |  |  |
| 571,00 | Cirrhosis hepatis cum alcoholism | 571.0 | Cirrhosis of liver, Alcoholic^4^ |
| 571,01 | Degeneratio hepatica adiposa alcoholic |  |  |
| AUD, alcohol use disorder; ICD, International Statistical Classification of Diseases and Related Health Problems; ICD-10, 10^th^ revision of the ICD; ICD-9, 9^th^ revision of the ICD; ICD-8, 8^th^ revision of the ICD.  This table adapted from Supplementary table 1 in Kane K, Westman J, Franck J, Gissler M. Risk of severe mood and anxiety disorders in the adult children of parents with alcohol use disorder: a nationwide cohort study. J Epidemiol Community Health. 2024 Jun 10;78(7):444-450. doi: 10.1136/jech-2023-221720. PMID: 38688702; PMCID: PMC11187371 under the terms of the CC BY-NC 4.0 license: <https://creativecommons.org/licenses/by/4.0/> [1]. The original table has been revised and expanded to provide more information about the international versions of the codes included in the variable, including English names and differences between the code systems used in Sweden and elsewhere.  Sources used to match disorders in the international and Swedish versions of the ICD-10 [2-6], ICD-9 [4, 7-13], and ICD-8 [4, 14-18] are shown in the reference list.  ^1^The international ICD-8 codes 261 Thiamine deficiency and 262 Niacin deficiency were not specifically related to alcohol use, but the Swedish equivalents were. Both Swedish codes were therefore included in the outcome variable.  ^2^The international ICD-8 code 291.9 Other and unspecified alcoholic psychoses was divided into two codes in Sweden: 1) 291,98 Psychosis alcoholica alia definite (Alcoholic psychosis, other specified) and 2) 291,99 Psychosis alcoholica nud (Alcoholic psychosis, unspecified). Both Swedish codes were included in the outcome variable.  ^3^The international ICD-8 code 303.9 Other and unspecified alcoholism was divided into two codes in Sweden: 1) 303,98 Alcoholismus alius definitus (Alcoholism, other specified) and 2) 303,99 Alcoholismus nud (Alcoholism, unspecified). Both Swedish codes were included in the outcome variable.  ^4^The international ICD-8 code 571.0 Cirrhosis of liver, Alcoholic was divided into two codes in Sweden: 1) 571,00 Cirrhosis hepatis cum alcoholism (Cirrhosis of the liver with alcoholism) and 2) 571,01 Degeneratio hepatica adiposa alcoholic (Alcoholic fatty degeneration of the liver). Both Swedish codes were included in the outcome variable. | | | |

| **Additional file 2.** ICD codes used to define AUD (ICD-10 code F10 and historical equivalents; the narrower definition of AUD used in the study) | | | |  |
| --- | --- | --- | --- | --- |
| **ICD-10** Mental and behavioral disorders due to use of alcohol | | | |  |
| F10.0 | Acute intoxication due to use of alcohol |  |  |  |
| F10.1 | Harmful use due to use of alcohol |  |  |  |
| F10.2 | Dependence syndrome due to use of alcohol |  |  |  |
| F10.3 | Withdrawal state due to use of alcohol |  |  |  |
| F10.4 | Withdrawal state with delirium due to use of alcohol |  |  |  |
| F10.5 | Psychotic disorder due to use of alcohol |  |  |  |
| F10.6 | Amnesic syndrome due to use of alcohol |  |  |  |
| F10.7 | Residual and late-onset psychotic disorder due to use of alcohol |  |  |  |
| F10.8 | Other mental and behavioral disorders due to use of alcohol |  |  |  |
| F10.9 | Unspecified mental and behavioral disorder |  |  |  |
| **ICD-9** |  |  |  |  |
| 291A | Delirium tremens | 291.0 | Delirium tremens |  |
| 291B | Korsakovs psykos, alkoholbetingad | 291.1 | Korsakov’s psychosis, alcoholic |  |
| 291C | Alkoholbetingad demens annan än 291A-B | 291.2 | Other alcoholic dementia |  |
| 291D | Alkoholhallucinos | 291.3 | Alcoholic hallucinosis |  |
| 291E | Patologiskt alkoholrus | 291.4 | Pathological drunkenness |  |
| 291F | Patologisk svartsjuka [alkohol relaterat] | 291.5 | Alcoholic jealousy (OR Pathological alcohol-related jealousy) |  |
| 291W | Specificerad alkoholpsykos annan än 291A-F | 291.8 | Other alcoholic psychosis |  |
| 291X | Alkoholpsykoser, ospecificerade | 291.9 | Alcoholic psychoses, unspecified |  |
| 305A | Alkoholmissbruk | 305.0 | Nondependent abuse of alcohol |  |
| 305X | Alkoholberoende | 303 | Alcohol dependence syndrome |  |
| **ICD-8** |  |  |  |  |
| 291,00 | Delirium tremens | 291.0 | Delirium tremens | |
| 291,10 | Psychosis korsakow (alcoholica) | 291.1 | Korsakov’s psychosis (alcoholic) | |
| 291,20 | Hallucinosis alcoholica alia | 291.2 | Other alcoholic hallucinosis | |
| 291,30 | Paranoia alcoholica | 291.3 | Alcoholic paranoia | |
| 291,98 | Psychosis alcoholica alia definita | 291.9 | Other and unspecified alcoholic psychoses^1^ | |
| 291,99 | Psychosis alcoholica nud |  |  |  |
| 303,00 | Alcoholismus episodicus | 303.0 | Episodic excessive drinking | |
| 303,10 | Alcoholismus habitualis | 303.1 | Habitual excessive drinking | |
| 303,20 | Alcoholismus chronicus ("alcoholic addiction") (dipsomania) | 302.2 | Alcohol addiction | |
| 303,98 | Alcoholismus alius definitus | 303.9 | Other and unspecified alcoholism^2^ | |
| 303,99 | Alcoholismus nud |  |  |  |
| AUD, alcohol use disorder; ICD, International Statistical Classification of Diseases and Related Health Problems; ICD-10, 10^th^ revision of the ICD; ICD-9, 9^th^ revision of the ICD; ICD-8, 8^th^ revision of the ICD.  Sources used to match disorders in the international and Swedish versions of the ICD-10, ICD-9 [4, 13], and ICD-8 [4, 18] over the course of the study period are shown in the reference list.  ^1^The international ICD-8 code 291.9 Other and unspecified alcoholic psychoses was divided into two codes in Sweden: 1) 291,98 Psychosis alcoholica alia definite (Alcoholic psychosis, other specified) and 2) 291,99 Psychosis alcoholica nud (Alcoholic psychosis, unspecified). Both Swedish codes were included in the outcome variable.  ^2^The international ICD-8 code 303.9 Other and unspecified alcoholism was divided into two codes in Sweden: 1) 303,98 Alcoholismus alius definitus (Alcoholism, other specified) and 2) 303,99 Alcoholismus nud (Alcoholism, unspecified). Both Swedish codes were included in the outcome variable. | | | |  |

| **Additional file 3.** ICD codes used to define opioid use disorder | | | |
| --- | --- | --- | --- |
| **ICD-10** | | | |
| F11.0 | Acute intoxication due to use of opioids |  |  |
| F11.1 | Harmful use due to use of opioids |  |  |
| F11.2 | Dependence syndrome due to use of opioids |  |  |
| F11.3 | Withdrawal state due to use of opioids |  |  |
| F11.4 | Withdrawal state with delirium due to use of opioids |  |  |
| F11.5 | Psychotic disorder due to use of opioids |  |  |
| F11.6 | Amnesic syndrome due to use of opioids |  |  |
| F11.7 | Residual and late-onset psychotic disorder due to use of opioids |  |  |
| F11.8 | Other mental and behavioral disorders due to use of opioids |  |  |
| F11.9 | Unspecified mental and behavioral disorder due to use of opioids |  |  |
| **ICD-9** | | | |
| 304A | Narkomani, morfin och andra opiater | 304.0 | Drug dependence, morphine type |
| **ICD-8** | | | |
| 304,00 | Narcomania, abusus medicamentorum / Opium, opii alcaloides et derivata ex iis | 304.0 | Drug dependence, Opium, opium alkaloids and their derivatives |
| 304,10 | Narcomania, abusus medicamentorum / Analgetica synthetica cum effectu simili morphino | 304.1 | Drug dependence, Synthetic analgesics with morphine-like effects |
| ICD, International Statistical Classification of Diseases and Related Health Problems; ICD-10, 10^th^ revision of the ICD; ICD-9, 9^th^ revision of the ICD; ICD-8, 8^th^ revision of the ICD.  Sources used to match opioid used disorder codes in the international and Swedish versions of the ICD-10 [4, 5, 8], ICD-9 [4, 12-14, 19, 20], and ICD-8 [4, 12, 14, 18] are shown in the reference list.  Only drug-specific ICD-8 and ICD-9 codes could be used in the analyses of specific substance use disorders. Some ICD-8 and ICD-9 codes were not drug-specific, and an additional code, unavailable to the researchers, would have been required to specify the drug involved. An example is Swedish ICD-9 code 305X Missbruk av droger och läkemedel. Although it is the equivalent of international ICD-9 code 305.5 Nondependent abuse of drugs, Morphine type, unlike the international code, it is not specific to morphine-type drugs. | | | |

| **Additional file 4.** ICD codes used to define cannabinoid use disorder | | | |
| --- | --- | --- | --- |
| **ICD-10** | | | |
| F12.0 | Acute intoxication due to use of cannabinoids |  |  |
| F12.1 | Harmful use due to use of cannabinoids |  |  |
| F12.2 | Dependence syndrome due to use of cannabinoids |  |  |
| F12.3 | Withdrawal state due to use of cannabinoids |  |  |
| F12.4 | Withdrawal state with delirium due to use of cannabinoids |  |  |
| F12.5 | Psychotic disorder due to use of cannabinoids |  |  |
| F12.6 | Amnesic syndrome due to use of cannabinoids |  |  |
| F12.7 | Residual and late-onset psychotic disorder due to use of cannabinoids |  |  |
| F12.8 | Other mental and behavioral disorders due to use of cannabinoids |  |  |
| F12.9 | Unspecified mental and behavioral disorder due to use of cannabinoids |  |  |
| **ICD-9** | | | |
| 304D | Narkomani, cannabis | 304.3 | Drug dependence, Cannabis |
| **ICD-8** | | | |
| 304,50 | Narcomania, abusus medicamentorum / Cannabis sativa | 304.5 | Drug dependence, Cannabis sativa |
| ICD, International Statistical Classification of Diseases and Related Health Problems; ICD-10, 10^th^ revision of the ICD; ICD-9, 9^th^ revision of the ICD; ICD-8, 8^th^ revision of the ICD.  Sources used to match cannabinoid use disorder codes in the international and Swedish versions of the ICD-10 [3-5], ICD-9 [4, 8, 12, 13, 19-21], and ICD-8 [4, 12, 14, 18] are shown in the reference list.  Only drug-specific ICD-8 and ICD-9 codes could be used in the analyses of specific substance use disorders. Some ICD-8 and ICD-9 codes were not drug-specific, and an additional code, unavailable to the researchers, would have been required to specify the drug involved. An example is Swedish ICD-9 code 305X Missbruk av droger och läkemedel. Although it is the equivalent of international ICD-9 code 305.2 Nondependent abuse of drugs, Cannabis, it is not specific to cannabis. | | | |

| **Additional file 5.** ICD codes used to define sedative or hypnotic use disorder | | | |
| --- | --- | --- | --- |
| **ICD-10** | | | |
| F13.0 | Acute intoxication due to use of sedatives or hypnotics |  |  |
| F13.1 | Harmful use due to use of sedatives or hypnotics |  |  |
| F13.2 | Dependence syndrome due to use of sedatives or hypnotics |  |  |
| F13.3 | Withdrawal state due to use of sedatives or hypnotics |  |  |
| F13.4 | Withdrawal state with delirium due to use of sedatives or hypnotics |  |  |
| F13.5 | Psychotic disorder due to use of sedatives or hypnotics |  |  |
| F13.6 | Amnesic syndrome due to use of sedatives or hypnotics |  |  |
| F13.7 | Residual and late-onset psychotic disorder due to use of sedatives or hypnotics |  |  |
| F13.8 | Other mental and behavioral disorders due to use of sedatives or hypnotics |  |  |
| F13.9 | Unspecified mental and behavioral disorder due to use of sedatives or hypnotics |  |  |
| **ICD-9** | | | |
| 304B | Narkomani, barbiturater | 304.1 | Drug dependence, Barbiturate type |
| **ICD-8** | | | |
| 304,20 | Narcomania, abusus medicamentorum / Barbituratum | 304.2 | Drug dependence, Barbiturates |
| 304,3 | Narcomania, abusus medicamentorum / Alia hypnotica et sedativa sive »tranquillizers» | 304.3 | Drug dependence, Other hypnotics and sedatives and tranquillizers |
| ICD, International Statistical Classification of Diseases and Related Health Problems; ICD-10, 10^th^ revision of the ICD; ICD-9, 9^th^ revision of the ICD; ICD-8, 8^th^ revision of the ICD.  Sources used to match sedative or hypnotic use disorder codes in the international and Swedish versions of the ICD-10 [3-5], ICD-9 [4, 8, 12, 13, 19, 21], and ICD-8 [4, 12, 14, 18] are shown in the reference list.  Only drug-specific ICD-8 and ICD-9 codes could be used in the analyses of specific substance use disorders. Some ICD-8 and ICD-9 codes were not drug-specific, and an additional code, unavailable to the researchers, would have been required to specify the drug involved. An example is Swedish ICD-9 code 305X Missbruk av droger och läkemedel. Although it is the equivalent of international ICD-9 code 305.4 Nondependent abuse of drugs, Barbiturates and tranquillizers, it is not specific to barbiturates and tranquilizers. | | | |

| **Additional file 6.** ICD codes used to define cocaine use disorder | | | |
| --- | --- | --- | --- |
| **ICD-10** | | | |
| F14.0 | Acute intoxication due to use of cocaine |  |  |
| F14.1 | Harmful use of cocaine |  |  |
| F14.2 | Dependence syndrome due to use of cocaine |  |  |
| F14.3 | Withdrawal state due to use of cocaine |  |  |
| F14.4 | Withdrawal state with delirium due to use of cocaine |  |  |
| F14.5 | Psychotic disorder due to use of cocaine |  |  |
| F14.6 | Amnesic syndrome due to use of cocaine |  |  |
| F14.7 | Residual and late-onset psychotic disorder due to use of cocaine |  |  |
| F14.8 | Other mental and behavioral disorders due to use of cocaine |  |  |
| F14.9 | Unspecified mental and behavioral disorder due to use of cocaine |  |  |
| **ICD-9** | | | |
| 304C | Narkomani, kokain | 304.2 | Drug dependence, Cocaine |
| **ICD-8** | | | |
| 304,4 | Narcomania, abusus medicamentorum / Cocainum | 304.4 | Drug dependence, Cocaine |
| ICD, International Statistical Classification of Diseases and Related Health Problems; ICD-10, 10^th^ revision of the ICD; ICD-9, 9^th^ revision of the ICD; ICD-8, 8^th^ revision of the ICD.  Sources used to match cocaine use disorder codes in the international and Swedish versions of the ICD-10 [3-5], ICD-9 [4, 8, 12, 19, 21], and ICD-8 [4, 12, 14, 18] are shown in the reference list.  Only drug-specific ICD-8 and ICD-9 codes could be used in the analyses of specific substance use disorders. Some ICD-8 and ICD-9 codes were not drug-specific, and an additional code, unavailable to the researchers, would have been required to specify the drug involved. An example is Swedish ICD-9 code 305X Missbruk av droger och läkemedel. Although it is the equivalent of international ICD-9 code 305.6 Nondependent abuse of drugs, Cocaine, it is not specific to cocaine. | | | |

| **Additional file 7.** ICD codes used to define other stimulant use disorder | | | |
| --- | --- | --- | --- |
| **ICD-10** | | | |
| F15.0 | Acute intoxication due to use of other stimulants, including caffeine |  |  |
| F15.1 | Harmful use due to use of other stimulants, including caffeine |  |  |
| F15.2 | Dependence syndrome due to use of other stimulants, including caffeine |  |  |
| F15.3 | Withdrawal state due to use of other stimulants, including caffeine |  |  |
| F15.4 | Withdrawal state with delirium due to use of other stimulants, including caffeine |  |  |
| F15.5 | Psychotic disorder due to use of other stimulants, including caffeine |  |  |
| F15.6 | Amnesic syndrome due to use of other stimulants, including caffeine |  |  |
| F15.7 | Residual and late-onset psychotic disorder due to use of other stimulants, including caffeine |  |  |
| F15.8 | Other mental and behavioral disorders due to use of other stimulants, including caffeine |  |  |
| F15.9 | Unspecified mental and behavioral disorder due to use of other stimulants, including caffeine |  |  |
| **ICD-9** | | | |
| 304E | Narkomani, amfetamin och andra psykostimulantia | 304.4 | Drug dependence, Amphetamine type and other psychostimulants (includes Phenmetrazine and Methylphenidate) |
| **ICD-8** | | | |
| 304,6 | Narcomania, abusus medicamentorum / Alia psychostimulantia | 304.6 | Drug dependence, Other psycho-stimulants |
| ICD, International Statistical Classification of Diseases and Related Health Problems; ICD-10, 10^th^ revision of the ICD; ICD-9, 9^th^ revision of the ICD; ICD-8, 8^th^ revision of the ICD.  Sources used to match other stimulant use disorder codes in the international and Swedish versions of the ICD-10 [3-5], ICD-9 [4, 8, 12, 13, 19, 21], and ICD-8 [4, 12, 14, 18] are shown in the reference list.  Only drug-specific ICD-8 and ICD-9 codes could be used in the analyses of specific substance use disorders. Some ICD-8 and ICD-9 codes were not drug-specific, and an additional code, unavailable to the researchers, would have been required to specify the drug involved. An example is Swedish ICD-9 code 305X Missbruk av droger och läkemedel. Although it is the equivalent of international ICD-9 code 305.7 Nondependent abuse of drugs, Amphetamine type, it is not specific to amphetamine-type drugs. | | | |

| **Additional file 8.** ICD codes used to define hallucinogen use disorder | | | |
| --- | --- | --- | --- |
| **ICD-10** | | | |
| F16.0 | Acute intoxication due to use of hallucinogens |  |  |
| F16.1 | Harmful use of hallucinogens |  |  |
| F16.2 | Dependence syndrome due to use of hallucinogens |  |  |
| F16.3 | Withdrawal state due to use of hallucinogens |  |  |
| F16.4 | Withdrawal state with delirium due to use of hallucinogens |  |  |
| F16.5 | Psychotic disorder due to use of hallucinogens |  |  |
| F16.6 | Amnesic syndrome due to use of hallucinogens |  |  |
| F16.7 | Residual and late-onset psychotic disorder due to use of hallucinogens |  |  |
| F16.8 | Other mental and behavioral disorders due to use of hallucinogens |  |  |
| F16.9 | Unspecified mental and behavioral disorder due to use of hallucinogens |  |  |
| **ICD-9** | | | |
| 304F | Narkomani, hallucinogener | 304.5 | Drug dependence, Hallucinogens |
| **ICD-8** | | | |
| 304,7 | Narcomania, abusus medicamentorum / Hallucinogenes | 304.7 | Drug dependence, Hallucinogens |
| ICD, International Statistical Classification of Diseases and Related Health Problems; ICD-10, 10^th^ revision of the ICD; ICD-9, 9^th^ revision of the ICD; ICD-8, 8^th^ revision of the ICD.  Sources used to match hallucinogen use disorder codes in the international and Swedish versions of the ICD-10 [3-5], ICD-9 [4, 8, 12, 13, 19, 21], and ICD-8 [4, 12, 14, 18] are shown in the reference list.  Only drug-specific ICD-8 and ICD-9 codes could be used in the analyses of specific substance use disorders. Some ICD-8 and ICD-9 codes were not drug-specific, and an additional code, unavailable to the researchers, would have been required to specify the drug involved. An example is Swedish ICD-9 code 305X Missbruk av droger och läkemedel. Although it is the equivalent of international ICD-9 code 305.3 Nondependent abuse of drugs, Hallucinogens, it is not specific to hallucinogens. | | | |

| **Additional file 9.** ICD codes used to define volatile solvent use disorder | | | |
| --- | --- | --- | --- |
| F18.0 | Acute intoxication due to use of volatile solvents |  |  |
| F18.1 | Harmful use of volatile solvents |  |  |
| F18.2 | Dependence syndrome due to use of volatile solvents |  |  |
| F18.3 | Withdrawal state due to use of volatile solvents |  |  |
| F18.4 | Withdrawal state with delirium due to use of volatile solvents |  |  |
| F18.5 | Psychotic disorder due to use of volatile solvents |  |  |
| F18.6 | Amnesic syndrome due to use of volatile solvents |  |  |
| F18.7 | Residual and late-onset psychotic disorder due to use of volatile solvents |  |  |
| F18.8 | Other mental and behavioral disorders due to use of volatile solvents |  |  |
| F18.9 | Unspecified mental and behavioral disorder due to use of volatile solvents |  |  |
| **ICD-9** | | | |
| 304G | Sniffning av thinner/bensin mm | 304.6 | Drug dependence, Other (includes glue sniffing)^1^ |
| **ICD-8** | | | |
| None | Not applicable^2^ | 304.8 | Other and combined drugs^2^ |
| ICD, International Statistical Classification of Diseases and Related Health Problems; ICD-10, 10^th^ revision of the ICD; ICD-9, 9^th^ revision of the ICD; ICD-8, 8^th^ revision of the ICD.  Sources used to match volatile solvent use disorder codes in the international and Swedish versions of the ICD-10 [3-5], ICD-9 [4, 8, 12, 13, 19, 21], and ICD-8 [4, 12, 14, 18] are shown in the reference list.  Only drug-specific ICD-8 and ICD-9 codes could be used in the analyses of specific substance use disorders. Some ICD-8 and ICD-9 codes were not drug-specific, and an additional code, unavailable to the researchers, would have been required to specify the drug involved. An example is Swedish ICD-9 code 305X Missbruk av droger och läkemedel, which is the equivalent of international ICD-9 code 305.9 Nondependent abuse of drugs, Other, mixed or unspecified. Neither the Swedish nor the international code is drug specific.  ^1^In the Swedish ICD-9, international code 304.6 Drug dependence, Other (includes glue sniffing) was split into two codes: 1) 304G Sniffning av thinner/bensin mm (Sniffing of thinner/gasoline etc.), and 2) 304W Narkomani, specificerade droger andra än 304A-H (Drug dependence, specified drugs other than 304A-H). Only 304G was included in the volatile solvent use disorder outcome.  ^2^In the Swedish ICD-8, volatile solvent use was given code 304,88 Narcomania, abusus medicamentorum / Alia definite (Narcomania, Drug abuse / Other defined), which is not drug specific. Because the research group did not have access to the code that specified the drug(s) associated with the diagnosis, ICD-8 code 304,88 was excluded from the analysis here and included in the analysis of multiple drug use and use of other psychoactive substances (Additional file 10). | | | |

| **Additional file 10.** ICD codes used to define multiple drug use and use of other psychoactive substances | | | |
| --- | --- | --- | --- |
| **ICD-10** | | | |
| F19.0 | Acute intoxication due to multiple drug use and use of other psychoactive substances |  |  |
| F19.1 | Harmful use due to multiple drug use and use of other psychoactive substances |  |  |
| F19.2 | Dependence syndrome due to multiple drug use and use of other psychoactive substances |  |  |
| F19.3 | Withdrawal state due to multiple drug use and use of other psychoactive substances |  |  |
| F19.4 | Withdrawal state with delirium due to multiple drug use and use of other psychoactive substances |  |  |
| F19.5 | Psychotic disorder due to multiple drug use and use of other psychoactive substances |  |  |
| F19.6 | Amnesic syndrome due to multiple drug use and use of other psychoactive substances |  |  |
| F19.7 | Residual and late-onset psychotic disorder due to multiple drug use and use of other psychoactive substances |  |  |
| F19.8 | Other mental and behavioral disorders due to multiple drug use and use of other psychoactive substances |  |  |
| F19.9 | Unspecified mental and behavioral disorders due to multiple drug use and use of other psychoactive substances |  |  |
| **ICD-9** | | | |
| 304H | Narkomani, kombination av olika droger | 304.7 | Drug dependence, Combinations of morphine type with any other^1^ |
|  |  | 304.8 | Drug dependence, Combinations excluding morphine type drug^1^ |
| 304W | Narkomani, andra specificerade droger | 304.6 | Drug dependence, Other (includes glue sniffing)^2^ |
| 304X | Narkomani, icke specificerade droger | 304.9 | Drug dependence, Unspecified |
| 648D | Andra aktuella tillstånd hos modern som klassificeras annorstädes men som uppträder som komplikation vid graviditet, barnsbörd och under barnsängstiden, Drogberoende tillstånd under 304 | 648.3 | Other current conditions in the mother classifiable elsewhere but complicating pregnancy,  childbirth, and the puerperium, Drug dependence |
| **ICD-8** | | | |
| 304,88 | Narcomania, abusus medicamentorum / Alia definita | 304.8 | Drug dependence, Other and combined drugs |
| 304,99 | Narcomania, abusus medicamentorum / non ultra descriptus | 304.9 | Drug dependence, Unspecified |
| ICD, International Statistical Classification of Diseases and Related Health Problems; ICD-10, 10^th^ revision of the ICD; ICD-9, 9^th^ revision of the ICD; ICD-8, 8^th^ revision of the ICD.  Sources used to match multiple drug use codes in the international and Swedish versions of the ICD-10 [3-5], ICD-9 [4, 8, 12, 13, 19, 21, 22], and ICD-8 [4, 12, 14, 18] over the course of the study period are shown in the reference list.  ^1^The international ICD-8 codes 304.7 Drug dependence, Combinations of morphine type with any other and 304.8 Drug dependence, Combinations excluding morphine type drug were combined into one code in Sweden, 304H Narkomani, kombination av olika droger (Drug dependence, combination of different drugs). The single Swedish code was included in the variable.  ^2^In the Swedish ICD-9, international code 304.6 Drug dependence, Other (includes glue sniffing) was split into two codes: 1) 304G Sniffning av thinner/bensin mm (Sniffing of thinner/gasoline etc.), and 2) 304W Narkomani, specificerade droger andra än 304A-H (Drug dependence, specified drugs other than 304A-H). Only 304W Drug dependence, specified drugs other than 304A-H was used in the multiple drug use outcome. | | | |

| **Additional file 11.** Hazard ratios and 95% confidence intervals of any, one, and two or more SUDs^1^ in the study population^2^ in sequentially adjusted models^3^ excluding vs. including AUD | | |
| --- | --- | --- |
| Composite variable | Excluding AUD (“any SUD excluding AUD”) | Including AUD (“any SUD”) |
| **Any SUD, HR (95% CI)** | | |
| Model 1 | 2.88 (2.71-3.06) | 3.22 (3.16-3.28) |
| Model 2 | 2.30 (2.16-2.45) | 2.65 (2.60-2.71) |
| Model 3 | 1.60 (1.49-1.72) | 1.89 (1.85-1.94) |
| Model 4 | 1.38 (1.28-1.48) | 1.68 (1.64-1.72) |
|  |  |  |
| **One SUD, HR (95% CI)** | | |
| Model 1 | 2.80 (2.63-2.99) | 2.58 (2.51-2.65) |
| Model 2 | 2.26 (2.11-2.42) | 2.22 (2.15-2.28) |
| Model 3 | 1.61 (1.49-1.74) | 1.76 (1.71-1.82) |
| Model 4 | 1.39 (1.29-1.51) | 1.61 (1.56-1.67) |
|  |  |  |
| **Two or more SUDs, HR (95% CI)** | | |
| Model 1 | 3.40 (2.92-3.97) | 4.39 (4.26-4.52) |
| Model 2 | 2.53 (2.16-2.97) | 3.38 (3.28-3.49) |
| Model 3 | 1.56 (1.29-1.88) | 2.09 (2.02-2.17) |
| Model 4 | 1.30 (1.07-1.57) | 1.77 (1.70-1.84) |
| AUD, alcohol use disorder; CI, confidence interval; HR, hazard ratio; ICD, International Statistical Classification of Diseases and Related Health Problems; ICD-10, 10th revision of the ICD; ICD-9, 9th revision of the ICD; ICD-8, 8th revision of the ICD; SUD, substance use disorder.  ^1^Any SUD: One or more ICD-10 codes F10-F16, F18-F19, as well as the ICD-8/ICD-9 equivalents; Any SUD excluding AUD: One or more ICD-10 codes F11-F16, F18, as well as the ICD-8/ICD-9 equivalents; One SUD: One ICD-10 code F10-F16 or F18 as well as the ICD-8/ICD-9 equivalents; One SUD excluding AUD: One ICD-10 code F11-F16, F18 as well as the ICD-8/ICD-9 equivalents; Two or more SUDs: F19 or two or more of ICD-10 codes F10-F16, F18 as well as the ICD-8/ICD-9 equivalents; Two or more SUDs excluding AUD: Two or more of ICD-10 codes F11-F16, F18, as well as the ICD-8/ICD-9 equivalents.  ^2^The 2,421,479 children born in Sweden (live births) between 1 January 1973 and 31 December 1995.  ^3^Model 1 included only severe parental AUD (yes/no). Model 2 was adjusted for the adult child’s sex, parental education, and death of at least one parent before the child turned 18 years. Model 3 was adjusted for the same factors as Model 2 plus parental SUD. Model 4 was adjusted for the same factors as Model 3 plus parental psychiatric disorder. | | |

| **Additional file 12**. Hazard ratios and 95% confidence intervals of one vs. two or more SUDs^1^ in the study population^2^ in sequentially adjusted models^3^ | | |
| --- | --- | --- |
|  | One SUD | Two or more SUDs |
| Including AUD, HR (95% CI) | | |
| Model 1 | 2.58 (2.51-2.65) | 4.39 (4.26-4.52) |
| Model 2 | 2.22 (2.15-2.28) | 3.38 (3.28-3.49) |
| Model 3 | 1.76 (1.71-1.82) | 2.09 (2.02-2.17) |
| Model 4 | 1.61 (1.56-1.67) | 1.77 (1.70-1.84) |
|  |  |  |
| Excluding AUD, HR (95% CI) | | |
| Model 1 | 2.80 (2.63-2.99) | 3.40 (2.92-3.97) |
| Model 2 | 2.26 (2.11-2.42) | 2.53 (2.16-2.97) |
| Model 3 | 1.61 (1.49-1.74) | 1.56 (1.29-1.88) |
| Model 4 | 1.39 (1.29-1.51) | 1.30 (1.07-1.57) |
| AUD, alcohol use disorder; CI, confidence interval; HR, hazard ratio; ICD, International Statistical Classification of Diseases and Related Health Problems; ICD-10, 10th revision of the ICD; ICD-9, 9th revision of the ICD; ICD-8, 8th revision of the ICD; SUD, substance use disorder.  ^1^One SUD: One ICD-10 code F10-F16 or F18 as well as the ICD-8/ICD-9 equivalents; One SUD excluding AUD: One ICD-10 code F11-F16, F18 as well as the ICD-8/ICD-9 equivalents; Two or more SUDs: F19 or two or more of ICD-10 codes F10-F16, F18 as well as the ICD-8/ICD-9 equivalents; Two or more SUDs excluding AUD: Two or more of ICD-10 codes F11-F16, F18, as well as the ICD-8/ICD-9 equivalents.  ^2^The 2,421,479 children born in Sweden (live births) between 1 January 1973 and 31 December 1995.  ^3^Model 1 included only severe parental AUD (yes/no). Model 2 was adjusted for the adult child’s sex, parental education, and death of at least one parent before the child turned 18 years. Model 3 was adjusted for the same factors as Model 2 plus parental SUD. Model 4 was adjusted for the same factors as Model 3 plus parental psychiatric disorder. | | |

**References**

1. Kane K, Westman J, Franck J, Gissler M: **Risk of severe mood and anxiety disorders in the adult children of parents with alcohol use disorder: a nationwide cohort study**. *J Epidemiol Community Health* 2024.

2. Swedish National Board of Health and Welfare [Socialstyrelsen]: **Instructions for coding of use and misuse of alcohol [Anvisningar för kodning av bruk och missbruk av alkohol]**; 2016.

3. **International Statistical Classification of Diseases and Related Health Problems 10th Revision (Browser)** [<https://icd.who.int/browse10/2019/en>]

4. van Drimmelen J: **The ICD-10 Classification of Mental and Behavioural Disorders: Conversion Tables between ICD-8, ICD-9 and ICD-10**. Geneva: Division of Mental Health of the World Health Organization; 1994.

5. Swedish National Board of Health and Welfare [Socialstyrelsen]: **International Statistical Classification of Diseases and Related Health Problems – Systematic list, Swedish version 2018 (ICD-10-SE) Part 1 A-G [Internationell statistisk klassifikation av sjukdomar och relaterade hälsoproblem – Systematisk förteckning, svensk version 2018 (ICD-10-SE)]**, 8th edn; 2021.

6. Swedish National Board of Health and Welfare [Socialstyrelsen]: **International Statistical Classification of Diseases and Related Health Problems – Systematic list, Swedish version 2018 (ICD-10-SE) Part 2 H-P [Internationell statistisk klassifikation av sjukdomar och relaterade hälsoproblem – Systematisk förteckning, svensk version 2018 (ICD-10-SE)]**, vol. 2 (3), 8th edn; 2021.

7. United States Centers for Medicare & Medicaid Services: **ICD-9-CM Diagnosis and Procedure Codes: Abbreviated and Full Code Titles Version 32 Full and Abbreviated Code Titles – Effective October 1, 2014 – Excel Spreadsheet**; 2014.

8. World Health Organization: **Chapter V: Mental Disorders, International Classification of Diseases Manual of the International Statistical Classification of Diseases, Injuries, and Causes of Death** vol. 1. Geneva; 1977.

9. World Health Organization and the International Conference for the Ninth Revision of the International Classification of Diseases: **Manual of the international statistical classification of diseases, injuries, and causes of death: based on the recommendations of the ninth revision conference, 1975, and adopted by the Twenty-ninth World Health Assembly**, 1975 revision. Geneva: World Health Organization; 1977.

10. **ICD-8 to ICD-9 Conversion Table** [<https://www.socialstyrelsen.se/>]

11. **Alcohol-Related Disease Impact (ARDI) International Classification of Diseases (ICD) Codes and Alcohol-Attributable Fraction (AAF) Sources** [<https://www.cdc.gov/alcohol/ardi/alcohol-related-icd-codes.html>]

12. Swedish National Board of Health and Welfare [Socialstyrelsen]: **Classification of Diseases 1987 Systematic List Swedish version of the International Classification of Diseases, Ninth Revision (ICD-9) (Foreword and introduction) [Klassifikation av sjukdomer 1987 Systematisk förteckning Svenskt version av International Classification of Diseases, Ninth Revision (ICD-9)]**. Stockholm Liber; 1986.

13. Swedish National Board of Health and Welfare [Socialstyrelsen]: **ICD-9 Classification of diseases 1987 (KS87) (1987-1996) codes and code text in Excel format [Klassifikation av sjukdomar 1987 (KS87) (1987-1996) kod och kodtext i Excelformat]**: Swedish National Board of Health and Welfare; 2019.

14. U.S. Department of Health Education and Welfare Public Health Service National Center for Health Statistics: **Eighth Revision International Classification of Diseases, Adapted for Use in the United States**, vol. 1, Tabular List. Washington, D.C.: U.S. Government Printing Office; ca. 1965.

15. **International Classification of Diseases, Revision 8 (1965)** [<https://www.meb.ki.se/svesan/ICD/icd8h.htm>]

16. **Appendix 1 - ICD 8 - Original.pdf. Adapted for use in the United States by U.S. Dept. of Health, Education, and Welfare, Public Health Service, National Center for Health Statistics. Originally adapted for the World Wide Web by the Comprehensive Epidemiologic Data Resource Project, Lawrence Berkeley National Laboratory;** n.d.

17. **ICD-9 to ICD-8 Conversion Table** [<https://www.socialstyrelsen.se>]

18. Swedish National Board of Health and Welfare [Socialstyrelsen]: **ICD-8 Classification of diseases etc. 1968 (1969-1986) codes and code text in Excel format [ICD-8 Klassifikation av sjukdomar m.m. 1968 (KS87) (1969-1986) kod och kodtext i Excelformat]**, vol. 2021: Swedish National Board of Health and Welfare; 2019.

19. Swedish National Board of Health and Welfare [Socialstyrelsen]: **Appendix: List of discrepancies between the 1987 Classification of Diseases and the International Classification of Diseases, Ninth Revision (ICD-9) [Bilaga. Förteckning över avvikelser mellan Klassifikation av sjukdomar 1987 och International Classification of Diseases, Ninth Revision (ICD-9)]**; n.d.

20. Swedish National Board of Health and Welfare [Socialstyrelsen]: **Classification of Diseases 1987 Systematic List Swedish version of the International Classification of Diseases, Ninth Revision (ICD-9) (full list) [Klassifikation av sjukdomer 1987 Systematisk förteckning Svensk version av International Classification of Diseases, Ninth Revision (ICD-9)]**; 1987.

21. Swedish National Board of Health and Welfare [Socialstyrelsen]: **Conversion table ICD-10 to ICD-9;** n.d.

22. World Health Organization: **Manual of the International Classification of Diseases, Injuries, and Causes of Death, Ninth Revision: Underlying and Multiple Cause of Death Codes For Deaths Occurring From 1979 - 1998**. Geneva; 1977.
